# Primary Care Utilization and Cardiovascular Screening in Adult Survivors of Childhood Cancer

**DOI:** 10.1001/jamanetworkopen.2023.47449

**Published:** 2023-12-13

**Authors:** Timothy J. D. Ohlsen, Yan Chen, Laura-Mae Baldwin, Melissa M. Hudson, Paul C. Nathan, Claire Snyder, Karen L. Syrjala, Emily S. Tonorezos, Yutaka Yasui, Gregory T. Armstrong, Kevin C. Oeffinger, Eric J. Chow

**Affiliations:** 1 Cancer and Blood Disorders Center, Seattle Children’s Hospital, University of Washington, Seattle; 2 Fred Hutchinson Cancer Center, Seattle, Washington; 3 University of Alberta, Calgary, Canada; 4 Department of Family Medicine, University of Washington, Seattle; 5 Departments of Oncology and Epidemiology and Cancer Control, St Jude Children’s Research Hospital, Memphis, Tennessee; 6 The Hospital for Sick Children, University of Toronto, Toronto, Canada; 7 Johns Hopkins School of Medicine, Baltimore, Maryland; 8 Office of Cancer Survivorship, Division of Cancer Control and Population Sciences, National Cancer Institute, Rockville, Maryland; 9 Department of Medicine, Duke University, Durham, North Carolina

## Abstract

**Importance:**

Cardiovascular disease is the leading noncancer cause of premature death among survivors of childhood cancer. Adult survivors of childhood cancer are largely managed by primary care practitioners (PCPs), and health care utilization patterns related to cardiovascular screening are not well described.

**Objective:**

To examine screening and health care utilization among survivors of childhood cancer at high risk for cardiovascular complications.

**Design, Setting, and Participants:**

This multicenter cross-sectional study included participants enrolled in a randomized clinical trial from 2017 to 2021. Abstracted documentation of participants’ cancer history, cardiotoxic treatment exposures, and survivorship care plans were obtained from participants’ PCPs spanning 2 years preceding trial enrollment. Participants were members of the Childhood Cancer Survivor Study cohort at elevated risk for ischemic heart disease or heart failure, enrolled in a randomized trial focused on improving cardiovascular risk factor control. Data were analyzed from November 2022 to July 2023.

**Main Outcomes and Measures:**

Outcomes of interest were numbers of PCP and specialist visits, cardiovascular risk factors (hypertension, dyslipidemia, and diabetes), risk factor screening, and cardiac testing. Multivariable logistic regression assessed characteristics associated with up-to-date cardiac testing at enrollment.

**Results:**

Of 347 enrolled participants, 293 (84.4%) had evaluable medical records (median [range] age, 39.9 [21.5-65.0] years; 149 [50.9%] male) and were included in analyses. At baseline, 238 participants (81.2%) had a documented PCP encounter; 241 participants (82.3%) had undergone blood pressure screening, 179 participants (61.1%) had undergone lipid testing, and 193 participants (65.9%) had undergone diabetes screening. A total of 63 participants (21.5%) had echocardiography completed or planned. Only 198 participants (67.6%) had records referencing a cancer history. PCP documentation of prior cardiotoxic exposures was low compared with known exposures, including radiation therapy (103 participants [35.2%] vs 203 participants [69.3%]; *P* < .001) and anthracycline chemotherapy (27 participants [9.2%] vs 222 participants [75.8%]; *P* = .008). Few records referenced a need for cancer-related late effects surveillance (95 records [32.4%]). Independent factors associated with cardiac screening included documentation of increased cardiovascular disease risk (odds ratio [OR], 11.94; 95% CI, 3.37-42.31), a late-effects surveillance plan (OR, 3.92; 95% CI, 1.69-9.11), and existing cardiovascular risk factors (OR per each additional factor, 2.09; 95% CI, 1.32-3.31).

**Conclusions and Relevance:**

This cross-sectional study of adult survivors of childhood cancer at increased risk of cardiovascular disease found low adherence to recommended cardiac testing and documentation of risk for these individuals. Improving accuracy of reporting of survivors’ exposures and risks within the medical record may improve screening.

## Key Points

**Question:**

How do adult survivors of childhood cancer use health care and cardiovascular screening with their primary care practitioners (PCPs)?

**Findings:**

In this cross-sectional study including 293 survivors of childhood cancer at high risk for cardiovascular complications, participants’ PCP records had infrequent documentation referencing a cancer history (67.6%) or a need for late-effects surveillance (32.4%), and only 21.5% of participants records had a completed or planned echocardiogram in the prior 2 years. Factors associated with up-to-date cardiac screening included documentation of increased cardiovascular risks or a late-effects surveillance plan.

**Meaning:**

These findings suggest that increasing participant and PCP awareness of risks and surveillance recommendations may improve adherence to screening.

## Teaser

This cross-sectional study examines screening and health care utilization rates among survivors of childhood cancer at high risk for cardiovascular complications.

## Introduction

With approximately 16 000 children in the US diagnosed with cancer annually and 5-year survival now exceeding 85%, an estimated half a million survivors of childhood cancer reside in the US.^1,2,3^ Among long-term survivors of childhood cancer, cardiovascular disease is the leading noncancer cause of premature death.^4,5,6,7^ Cancer-related treatment exposures place survivors at up to 5-fold increased risk for cardiovascular disease and related death.^4,5,7^ Importantly, modifiable cardiovascular risk factors, such as hypertension, dyslipidemia, and diabetes, are more prevalent among survivors of cancer compared with the general population, and these risk factors contribute a proportionally greater impact on cardiovascular mortality compared with their impact on the general population.^6,7^

Screening for and managing these risk factors following cancer therapy may reduce long-term morbidity and mortality from cardiovascular disease.^8^ Multiple guidelines exist for survivors of childhood cancer, particularly those at increased risk of heart disease due to prior treatment exposures.^9,10,11,12,13^ These guidelines recommend regular screening for hypertension, dyslipidemia, and diabetes. Early detection of anthracycline-associated cardiomyopathy using surveillance echocardiography or equivalent modalities has also been recommended. Since most adult survivors of childhood cancer are treated exclusively by primary care practitioners (PCPs) without involvement from oncologists (pediatric or otherwise) or regular visits to a cancer survivorship clinic, effective cardiovascular care requires engaged PCPs who are knowledgeable about these risks.^14,15,16,17^ However, little is known about health care utilization patterns in this phase of survivorship, including the testing for and prevention of cardiovascular disease.

As part of a randomized intervention trial to improve control of cardiovascular risk factors among long-term survivors of cancer,^18^ we examined cardiovascular screening and health care utilization patterns among survivors of childhood cancer at high risk for cardiovascular complications. To achieve this, we comprehensively reviewed medical record information from participants’ PCPs, covering the 2-year period leading up to trial enrollment, with the goal of identifying factors associated with cardiac screening. Furthermore, as many survivors of childhood cancer lack critical knowledge regarding their own treatment exposures and risk for late effects,^19,20,21^ we compared the accuracy of PCP records and survivor self-report against research records from survivors’ original cancer treatment institutions. Deficits in both PCPs’ and survivors’ knowledge can highlight important targets to improve communication among oncology, survivorship, and primary care teams to increase adherence to recommended screening.

## Methods

This was a cross-sectional study of adult survivors of childhood cancer enrolled in a randomized trial to promote control of cardiac risk factors. Study procedures were approved by the Fred Hutchinson Cancer Center and St Jude Children’s Research Hospital institutional review boards. All participants provided written informed consent for data collection. This report follows the Strengthening the Reporting of Observational Studies in Epidemiology (STROBE) reporting guideline for cross-sectional studies.

### Participants

The Childhood Cancer Survivor Study (CCSS) is an ongoing multi-institutional cohort study including approximately 25 700 five-year survivors of the most common types of childhood cancer (leukemia, lymphoma, renal tumors, sarcomas, and central nervous system malignant neoplasms) diagnosed before age 21 years between 1970 and 1999. The CCSS has abstracted cancer treatment exposures within 5 years of diagnosis from treating institutions’ records, including radiation therapy, chemotherapy, and surgical procedures.^22^ The Communicating Health Information and Improving Coordination With Primary Care study (CHIIP) is a randomized clinical trial conducted within CCSS to test the efficacy of a personalized survivorship care plan (SCP) intervention tailored to improve control (ie, reduce undertreatment) of hypertension, dyslipidemia, and diabetes.^18^ For CHIIP, eligible participants were members of the CCSS cohort estimated to be at elevated risk for future cardiovascular disease, defined as an approximately 10% or greater risk of ischemic heart disease or heart failure by age 50 years, based on risk models incorporating demographic and cancer treatment exposures.^23,24^ We excluded participants with a history of known ischemic heart disease or heart failure at enrollment. Interested participants underwent a home visit by a trained examiner recruited between September 2017 and April 2021. Individuals found to have 1 or more potentially underdiagnosed (ie, previously unknown diagnosis) or undertreated (ie, known diagnosis but measured values out of the therapeutic reference range) cardiovascular risk factors (ie, hypertension, dyslipidemia, and glucose intolerance) were then randomized 1:1 into an intervention or a control group, and their PCP medical records were requested. Details on study eligibility and measurements have been published elsewhere.^18^ Randomized participants who were unable or unwilling to sign a Health Insurance Portability and Accountability Act waiver granting release of medical records and individuals whose PCP’s office could not or would not provide medical records were excluded from this analysis.

### Data Collection and Variables

At the time of consent, we asked all participants to list their PCPs over the 2 years preceding trial enrollment. We then requested PCP outpatient clinic medical records over this time frame, including clinician notes, medication lists, laboratory results, and imaging reports for all participants. Through manual record review, we ascertained the number of PCP and specialist visits, as well as documentation of the testing for, diagnosis of, and treatment of hypertension, dyslipidemia, and diabetes. We determined whether there was documentation of participants’ cancer history, cardiotoxic treatment exposures (ie, anthracycline-based chemotherapy, radiation therapy affecting the heart), need for late-effects screening, and the presence of any SCP, the latter of which we defined as a note or document providing a summary of a participant’s past cancer treatment combined with an evidence-based follow-up plan. We also documented whether more in-depth cardiac testing (eg, electrocardiogram [ECG], echocardiogram, or other imaging) was performed or planned.

On trial enrollment, we asked participants to self-report their personal medical history to allow for comparisons with CCSS research records and PCP medical record documentation. Specifically, participants were asked whether they had previously received radiation or cardiotoxic chemotherapy; had undergone various medical and cardiovascular testing in the preceding 2 years (ie, echocardiogram, blood pressure measurement, diabetes screening or testing, lipid screening or testing); or were using medications for hypertension, diabetes, or dyslipidemia. Participants’ historical treatment exposures were derived from CCSS research data, which were based on an abstraction of each survivor’s original cancer treatment records. Participant demographics were obtained from CCSS research data. Participants self-reported race and ethnicity to the CCSS study team. Race was categorized as American Indian or Alaska Native, Asian, Black, Pacific Islander, White, or unknown. Ethnicity was categorized as Hispanic or Latino, not Hispanic or Latino, or unknown. Other race and ethnicity categories were self-reported as other and nonspecified. Race and ethnicity were assessed to examine the study cohort and consider the generalizability of its findings to other populations. CCSS research data were considered the criterion standard in comparisons between different reporting methods.

### Statistical Analysis

Participant demographics, diagnosis characteristics, and historical treatment information were analyzed with descriptive statistics. We calculated the proportion of participants with medical visits and other health care utilization and cardiovascular screening based on review of the PCPs’ medical records. Medical record documentation of prior treatment exposures was compared with known exposures from CCSS research data by McNemar test. To assess agreement among participant self-report, PCP medical records, and CCSS research data, we estimated the sensitivity, specificity, and κ (concordance) among these data sources.

Univariate logistic regression models identified factors associated with having completed any cardiac screening (ECG, echocardiogram, or other cardiac imaging) in the 2 years preceding trial enrollment, with odds ratios (ORs) and 95% CIs. Variables of interest included age, sex, documentation of radiation and cardiotoxic chemotherapy exposures, documentation that the participant had increased risk of cardiovascular disease, presence of an SCP, documentation of the need for late-effects monitoring, number of existing cardiovascular conditions (as determined by PCP medical records), a recent cardiology visit, and greater PCP utilization (defined as having >3 PCP visits over the preceding 2 years; 3 being the median value). Variables with *P* < .20 in univariate testing were then included in a multivariable logistic regression model. *P* values were 2-sided, and statistical significance was set at *P* = .05. Statistical analysis was performed with SAS version 9.4 (SAS Institute). Data were analyzed from November 2022 to July 2023.

## Results

### Participant Characteristics

**Figure 1.**
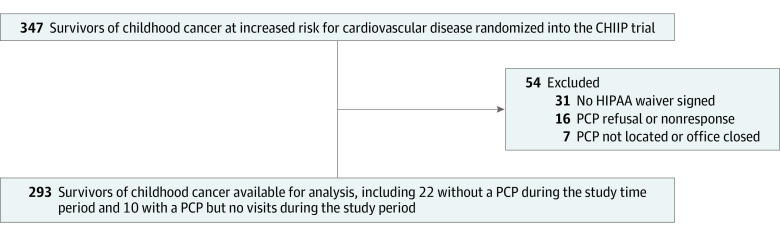
Selection Flowchart of Communicating Health Information and Improving Coordination With Primary Care (CHIIP) Trial Participants for Analysis HIPAA indicates Health Insurance Portability and Accountability Act; PCP, primary care practitioner.

Of 347 enrolled CHIIP participants, 293 (median [range] age, 39.9 [21.5-65.0] years; 149 [50.9%] male) had evaluable data for baseline health care utilization analysis (Figure 1 and Table 1). No participants had missing data for demographic variables of interest. The median (range) age of childhood cancer diagnosis was 9.3 (0.0-20.9) years. Compared with the overall CCSS cohort who completed the most recent survey (follow-up 5, conducted between 2014 and 2016), CHIIP study participants had similar demographic characteristics, including approximately 90% with self-reported health insurance and 90% having reported receiving routine medical care within the past 2 years (Table 1). By design, CHIIP participants were selected for individuals who had a diagnosis of hypertension, dyslipidemia, or diabetes.

**Table 1.** Demographic and Clinical Characteristics of CHIIP Study Participants Compared With the Overall CCSS Cohort

Characteristics	Participants, No. (%)	
	CHIIP (n = 293)	CCSS (n = 11 337)^a^
Age, median (range) at assessment, y	39.9 (21.5-65.0)	36.8 (16.0-65.9)
Sex		
Male	149 (50.9)	5419 (47.8)
Female	144 (49.2)	5918 (52.2)
Race		
American Indian or Alaska Native	0	55 (0.5)
Asian	5 (1.7)	160 (1.6)
Black	7 (2.4)	498 (4.6)
Pacific Islander	2 (0.7)	10 (0.1)
White	267 (91.1)	10195 (89.3)
>1 Race or other^b^	12 (4.1)	318 (3.1)
Unknown	0	101 (0.9)
Ethnicity		
Hispanic or Latino	23 (7.8)	710 (6.5)
Not Hispanic or Latino	270 (92.2)	10537 (92.7)
Unknown	0	90 (0.9)
Age at diagnosis, median (range), y	9.3 (0.0-20.9)	7.3 (0.0-21.0)
Cancer diagnosis		
Bone cancer	36 (12.3)	951 (7.4)
Central nervous system tumor	23 (7.9)	2000 (15.5)
Hodgkin lymphoma	60 (20.5)	1380 (10.7)
Kidney tumor	32 (10.9)	1070 (8.3)
Leukemia	94 (32.1)	3384 (38.4)
Non-Hodgkin lymphoma	23 (7.9)	936 (7.2)
Neuroblastoma	14 (4.8)	843 (6.5)
Soft tissue sarcoma	11 (3.8)	773 (6.0)
Cancer treatment exposures^c^		
Anthracycline chemotherapy	222 (75.8)	8689 (83.0)
Any radiation	203 (69.3)	5906 (50.8)
Self-reported health care insurance	273 (93.2)	10 477 (92.6)
Self-reported routine care visit within past 2 y	262 (89.4)	10 324 (91.3)
Self-reported history of diagnosed cardiovascular conditions		
Hypertension	94 (32.1)	2019 (16.3)
Dyslipidemia	109 (37.2)	2395 (19.9)
Diabetes	34 (11.6)	659 (5.6)
Prediabetes	22 (7.5)	498 (4.1)

Abbreviations: CCSS, Childhood Cancer Survivor Study; CHIIP, Communicating Health Information and Improving Coordination With Primary Care.

^a^ Members of the cohort who responded to the follow-up 5 survey, conducted between 2014 and 2016.

^b^ Includes those who self-reported as other or nonspecified.

^c^ Per CCSS records.

### Baseline Utilization of General Health Care and Cardiovascular Disease Screening

In the 2 years leading up to study enrollment, 238 participants (81.2%) had a documented PCP office visit (median [IQR], 3 [2-5] visits), and 63 participants (21.5%) had a subspecialty visit noted, including 12 participants (4.1%) with records documenting a cardiology specialty visit. Of 293 participants with evaluable data, 46 participants (15.7%) did not have any documented outpatient medical visits. A total of 241 participants (82.3%) had a blood pressure measurement, 179 participants (61.1%) had undergone lipid testing, and 193 participants (65.9%) had undergone diabetes testing in the preceding 2 years. Cardiac testing was performed in the prior 2 years for 85 evaluable participants (29.0%), including 63 participants (21.5%) with echocardiography, although all participants were eligible for guideline-recommended screening. According to self-report, 279 participants (95.2%) received blood pressure measurements, 235 participants (80.2%) had undergone lipid testing, 207 participants (70.7%) had undergone diabetes testing, and 114 participants (38.9%) had undergone echocardiography in the previous 2 years.

### Medical Record Documentation of Cancer History and Cardiovascular Risks

**Figure 2.**
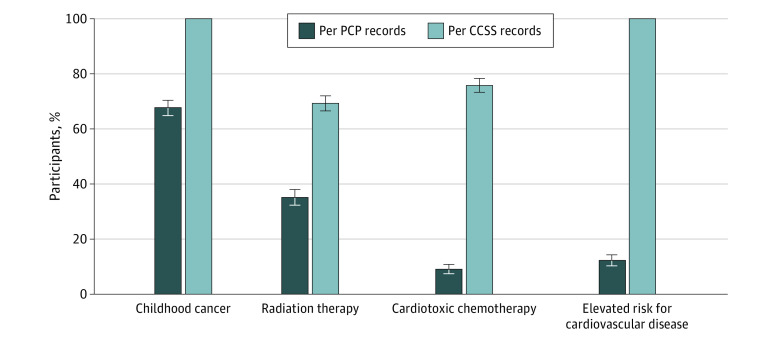
Frequency of Documentation of Treatment Exposures by Primary Care Provider (PCP) Records vs Data Abstracted From Childhood Cancer Survivor Study (CCSS) Research Records Elevated risk for cardiovascular disease was defined as an estimated 10% or greater risk of ischemic heart disease or heart failure by age 50 years, based on existing models.^23,24^

Although all participants had a history of childhood cancer, only 198 participants (67.6%) had any reference to a history of cancer noted in their medical records (vs 100% in the CCSS research records) (*P* < .001) (Figure 2). The frequency with which treatment exposures or risks were documented in the medical record was also significantly lower than those recorded in CCSS research data: radiation therapy (103 participants [35.2%] vs 203 participants [69.3%]; *P* < .001) and anthracycline chemotherapy (27 participants [9.2%] vs 222 participants [75.8%]; *P* < .001). Additionally, 95 participants (32.4%) had a documented need for late-effects surveillance (*P* < .001), and 14 participants (4.8%) had records outlining or referencing an SCP.

Concordance among participants’ self-report, primary care office documentation, and CCSS research data varied with regard to reported histories and cardiovascular screening (Table 2). In general, the specificity of PCP records related to treatment exposures was high, each greater than 95%, but sensitivity was low (49.3% for radiation history and 11.7% for receipt of cardiotoxic chemotherapy) (Table 2). The specificity of participant report compared with CCSS data was lower than PCP records, and sensitivity was greater for a history of radiation (96.1%) than for a history of cardiotoxic chemotherapy (44.6%) (Table 2). Concordance between participant report and PCP records was also low for radiation (κ = 0.31) and cardiotoxic chemotherapy κ = 0.13) exposures. We found strong concordance between self-report and PCP records with regard to hypertension, lipid, and diabetes medications (κ range 0.79-0.89), but poor concordance with regard to echocardiography (κ = 0.37) and other common cardiometabolic screening tests (κ range, 0.05-0.34).

**Table 2.** Sensitivity, Specificity, and Agreement Among Self-Report, PCP Records, and CCSS Research Records

Measure	Records		κ
	Sensitivity	Specificity	
**PCP records vs CCSS records, No./No. in CCSS (%)**			
History of radiation	100/203 (49.3)	87/90 (96.7)	0.35
History of cardiotoxic chemotherapy	26/222 (11.7)	70/71 (98.6)	0.05
**Self-report vs CCSS records, reported No./No. in CCSS (%)**			
History of radiation	195/203 (96.1)	76/90 (84.4)	0.82
History of cardiotoxic chemotherapy	99/222 (44.6)	66/71 (93.0)	0.24
**Self-report vs PCP records, reported No./No. of PCP records (%)**			
Medication use			
Hypertension	67/89 (75.3)	201/204 (98.5)	0.79
Diabetes	28/32 (87.5)	259/261 (99.2)	0.89
Lipid	49/53 (92.5)	234/240 (97.5)	0.89
Measurements or tests			
Blood pressure	231/241 (95.9)	4/52 (7.7)	0.05
Lipid screening	155/168 (92.3)	45/125 (36)	0.30
Diabetes screening	144/184 (78.3)	46/109 (42.2)	0.21
Echocardiogram	44/57 (77.2)	166/236 (70.3)	0.34

Abbreviations: CCSS, Childhood Cancer Survivor Study; PCP, primary care practitioner.

### Factors Associated With Completed or Planned Cardiac Testing

In univariate and multivariable logistic regression analysis, we identified several factors associated with having completed or planned cardiac testing at initial trial enrollment (Table 3). In the univariate model, factors associated with cardiac testing included documentation of radiation therapy exposure, documentation of cardiotoxic chemotherapy exposure, presence of existing cardiovascular risk factors in the medical record, the presence of an SCP, documentation of a need for late-effects surveillance, a cardiology visit in the prior 2 years, number of existing cardiovascular disease risk factors, and high PCP utilization (Table 3). In the multivariable model, documentation of a participant’s increased cardiovascular disease risk (OR, 11.94; 95% CI, 3.37-42.31), documentation of need for late-effects surveillance (OR, 3.92; 95% CI, 1.69-9.11), and the presence of existing cardiovascular risk factors in the medical record (OR, 2.09; 95% CI, 1.32-3.31) were independently associated with greater odds of having recent or planned cardiac screening.

**Table 3.** Factors Associated With Having Cardiac Testing Performed or Planned Among Adult Survivors of Childhood Cancer^a^

Characteristic	Participants with testing, No./No. evaluable (%) (n = 248)		Odds ratio (95% CI)^b^	
	Factor present	Factor absent	Univariate model	Multivariable model
Female sex	45/124 (36.3)	40/124 (32.3)	0.83 (0.50-1.39)	NA^c^
Current age	NA^d^	NA^d^	1.03 (1.00-1.05)	1.03 (0.99-1.07)
Documentation of radiation exposure	45/103 (43.7)	40/145 (27.6)	2.04 (1.19-3.47)	0.44 (0.18-1.06)
Documentation of cardiotoxic chemotherapy exposure	20/27 (74.1)	65/221 (29.4)	6.86 (2.77-17.00)	1.77 (0.48-6.51)
Documentation of increased CV disease risk	31/36 (86.1)	54/212 (25.5)	18.14 (6.71-49.01)	11.94 (3.37-42.31)
Survivorship care plan noted	11/14 (78.6)	74/234 (31.6)	7.93 (2.15-29.26)	3.96 (0.58-27.18)
Documentation of a cancer-related late-effects surveillance or follow–up plan	51/95 (53.7)	34/153 (22.2)	4.06 (2.33-7.07)	3.92 (1.69-9.11)
Existing CV risk factors, No.^e^	NA^d^	NA^d^	2.10 (1.49-2.95)	2.09 (1.32-3.31)
Cardiology visit during preceding 2 y	11/12 (91.7)	74/236 (31.4)	27.47 (3.49-216.37)	8.29 (0.90-79.32)
>3 PCP visits at baseline^f^	52/116 (44.8)	33/132 (25.0)	3.00 (1.77-5.11)	1.78 (0.87-3.65)

Abbreviations: CV, cardiovascular; NA, not applicable; PCP, primary care practitioner.

^a^ Cardiac testing was defined as electrocardiogram, echocardiogram, or other cardiac imaging.

^b^ Participants with missing variables were excluded from the regression model.

^c^ Sex was not significant in univariate testing (*P* = .49) and therefore was excluded from the multivariable model.

^d^ NA with nonbinary covariate.

^e^ Diagnosis of hypertension, dyslipidemia, or diabetes at baseline, modeled ordinally as 0, 1, and 2 or more conditions.

^f^ Based on median of 3 visits.

## Discussion

In this cross-sectional study of the PCP-documented screening patterns of a cohort of survivors of childhood cancer at high risk for cardiovascular disease, we found that adherence to regular cardiovascular screening was suboptimal. Few PCP records mentioned items pertinent to survivors’ cancer histories or a need for late-effects surveillance. Almost one-third of evaluable participants had no documentation acknowledging a history of childhood cancer. Furthermore, concordance between participant self-report and PCP records, compared with known exposures abstracted from original oncology treatment records, varied considerably but was often poor. Only 21.5% of these individuals at elevated risk of cardiovascular disease had recommended echocardiography planned or performed in the prior 2 years. This was similar to the rate identified from an earlier CCSS intervention study focused on enhancing cardiomyopathy screening.^25^ National guidelines specific to long-term survivors of childhood cancer that were in place during this time period recommended consideration of echocardiograms or similar screening every 1 to 2 years for these individuals.^10,13^ In comparison, basic screening for hypertension, dyslipidemia, and diabetes was more prevalent, perhaps reflective of the frequency of routine screening for these conditions in the general adult population without a cancer history.^26,27^ We observed low rates of SCP utilization, consistent with participants who were diagnosed before SCPs were recommended and received much of their survivorship care in an era generally marked by slow adoption among PCPs.^28^ Factors associated with having up-to-date cardiovascular disease screening included documentation in the medical record of a participant having an increased risk for cardiovascular disease and a need for late-effects surveillance.

Although several prior studies have raised concerns for suboptimal screening for multiple conditions among survivors of childhood cancer,^29,30,31^ no study to our knowledge has examined screening frequency and adherence through a direct review of PCP medical records. Therefore, we present survivors’ health behaviors from a unique perspective that complements participants’ self-report, which we elicited concurrently. The discordance that we observed between participants’ self-report and medical records with regard to certain treatment exposures agrees with prior investigations among adult survivors of childhood cancer demonstrating limited recall^19,20,21^ and emphasizes the importance of data collection from multiple sources. While some treatment exposures, such as radiation therapy, were reported by participants with reasonable accuracy, neither participants nor PCP medical records reliably reported anthracycline chemotherapy exposure. These discrepancies in both self-report and PCP medical records build on prior research that only assessed the accuracy of patient self-report and may highlight information to prioritize in communications from oncology and survivorship teams to patients and their PCPs. Our results cannot distinguish whether the act of documentation itself increased cardiac screening or was more reflective of a general awareness of risks for late effects. However, given that individual patients may be covered by multiple clinicians in a practice, documentation of risk by 1 clinicians could influence other clinicians’ actions at follow-up, regardless of general awareness regarding surveillance needs.

Although the overall adherence to cardiovascular screening recommendations among this selected population is disconcerting, our findings provide hope that streamlined communication between oncologists, patients, and primary care could improve awareness, adherence, and overall cardiovascular outcomes. As strategies emerge to prevent or treat anthracycline-associated cardiomyopathy, either through the reduction of modifiable disease risk factors or cardiac remodeling as heart function begins to worsen,^6,32,33^ multimodal strategies to address guideline-concordant survivorship care are needed. The CHIIP intervention trial in progress focuses on providing personalized SCPs to patients and PCPs.^18^ This strategy aims to address knowledge and communication gaps that have previously represented barriers to uptake.^18,31^ Combined with other initiatives, such as those designed to promote general awareness of survivorship care, increase access to survivorship services, reduce financial barriers to care, and address critical disparities,^17,21,25,34^ the CHIIP study’s intervention to systematically outline survivors’ individualized cardiovascular risks and other surveillance needs may improve adherence to recommended guidelines. Initial results have identified that survivors of cancer are twice as likely to have an undertreated cardiovascular risk factor compared with a matched general population sample.^35^ Ultimately, a multimodal strategy consisting of interventions to promote SCPs in tandem with increased PCP engagement, targeted counseling, sustainable community partnerships, and technology-based solutions will likely be most effective.^25,34,36^ For example, these strategies have shown improvement in cardiomyopathy screening with echocardiograms in adult survivors of childhood cancer from approximately 20% to 50%.^25^

### Limitations

There are important limitations to our study. Although our abstraction of available PCP records was comprehensive, it is possible that we did not capture all health care utilization. While PCPs were identified directly from study participants, it is possible that some screening and care related to cancer survivorship may have occurred at other sites not indicated by participants and not captured in the record abstraction. It is also conceivable that PCP offices did not receive or save all records from specialist visits or the results of studies ordered by specialists. Nevertheless, in this relatively young adult population who had to be free of serious cardiovascular disease due to the CHIIP study’s eligibility requirements, nearly a one-fourth did see a specialist in the prior 2 years and 4.1% had a documented cardiologist visit. A greater proportion of survivors reported having completed echocardiography (38.9%) in the past 2 years compared with those whose records indicated this (21.5%); however, participants’ responses could have been affected by recall bias, especially when asked about a specific 2-year time frame. Regardless, as PCPs likely manage overall screening cadences and order most tests, the lack of documentation in PCP records was concerning.

Our study cohort also comprises a large number of participants who identified as non-Hispanic White and were more likely to have health insurance than the general US population, among whom the proportions of uninsured adults aged 19 to 64 years ranges from 9% to 15%.^37^ Thus, analysis of this relatively privileged group potentially overestimates adherence among all long-term survivors of childhood cancer, given reported disparities in accessing various aspects of survivorship care.^38,39,40^ If so, these findings demonstrate an even greater need for solutions to optimize cardiovascular screening for all adult survivors of childhood cancer.

## Conclusions

In this cross-sectional study, adult survivors of childhood cancer at elevated risk for cardiovascular disease had low adherence to recommended cardiac testing and documentation of their risk based on comprehensive review of their primary care offices’ medical records. Acknowledgment of a participant’s increased risk for cardiovascular disease and documentation of a need for late-effects surveillance in the medical record were both independently associated with receipt of cardiac screening at the time of study enrollment. Increasing patients’ and PCPs’ awareness of cardiovascular risks related to cancer therapy and recommendations for surveillance may improve adherence to guideline-recommended care to detect and intervene on cardiovascular disease earlier to impact overall outcomes.

## Notes

Supplement.
Data Sharing Statement
