# Supplementary material for: Primary Care Utilization and Cardiovascular Screening in Adult Survivors of Childhood Cancer
Source: JAMA Netw Open. 2023 Dec 13;6(12):e2347449. doi: 10.1001/jamanetworkopen.2023.47449 (PMC10719759; doi:10.1001/jamanetworkopen.2023.47449)
Supplement: Supplement. — Data Sharing Statement [file jamanetwopen-e2347449-s001.pdf]

Data Sharing Statement

Ohlsen. Primary Care Utilization and Cardiovascular Screening in Adult Survivors of Childhood Cancer. *JAMA Netw Open*. Published December 13, 2023.
doi:10.1001/jamanetworkopen.2023.47449

Data
Data available: No
